# Deciphering Authentic Nociceptive Thalamic Responses in Rats

**DOI:** 10.34133/research.0348

**Published:** 2024-04-09

**Authors:** Zhenjiang Li, Libo Zhang, Fengrui Zhang, Lupeng Yue, Li Hu

**Affiliations:** ^1^ CAS Key Laboratory of Mental Health, Institute of Psychology, Chinese Academy of Sciences, 100101 Beijing, China.; ^2^ Department of Psychology, University of Chinese Academy of Sciences, 100049 Beijing, China.

## Abstract

The thalamus and its cortical connections play a pivotal role in pain information processing, yet the exploration of its electrophysiological responses to nociceptive stimuli has been limited. Here, in 2 experiments we recorded neural responses to nociceptive laser stimuli in the thalamic (ventral posterior lateral nucleus and medial dorsal nucleus) and cortical regions (primary somatosensory cortex [S1] and anterior cingulate cortex) within the lateral and medial pain pathways. We found remarkable similarities in laser-evoked brain responses that encoded pain intensity within thalamic and cortical regions. Contrary to the expected temporal sequence of ascending information flow, the recorded thalamic response (N1) was temporally later than its cortical counterparts, suggesting that it may not be a genuine thalamus-generated response. Importantly, we also identified a distinctive component in the thalamus, i.e., the early negativity (EN) occurring around 100 ms after the onset of nociceptive stimuli. This EN component represents an authentic nociceptive thalamic response and closely synchronizes with the directional information flow from the thalamus to the cortex. These findings underscore the importance of isolating genuine thalamic neural responses, thereby contributing to a more comprehensive understanding of the thalamic function in pain processing. Additionally, these findings hold potential clinical implications, particularly in the advancement of closed-loop neuromodulation treatments for neurological diseases targeting this vital brain region.

## Introduction

Animals can only live a short life without the ability to sense and respond to noxious stimuli. To detect and react to bodily damages promptly, pain conveys both sensory-discriminative and affective-motivational information [1,2]. These 2 types of pain information are typically transmitted via the lateral and medial pain pathways respectively. A key structure in these 2 pathways is the thalamus, the relay hub that receives nociceptive inputs from the spinal cord and forwards them to the cortex for further processing [3–6]. Specifically, the ventral posterior lateral nucleus (VPL) of the thalamus and its projections to the cortex such as the primary somatosensory cortex (S1) constitute the lateral pain pathway responsible for processing sensory-discriminative information of pain [7]. On the other hand, the medial dorsal nucleus (MD) of the thalamus and its projections to areas like the anterior cingulate cortex (ACC) comprise the medial pain pathway that processes the affective-motivational aspect of pain [8]. Notably, the MD is increasingly recognized as a potential target for pain treatment. Various thalamic nuclei, such as the centromedian-parafascicular nucleus, the periventricular gray, and the VPL, are frequently used as effective targets in deep brain stimulation to treat chronic pain [9]. Yet, existing studies indicate that the analgesic outcomes from the oft-chosen sensory thalamic targets fall short of expectations in some individuals [10,11]. The intervention of targets responsible for the emotional aspect of pain, such as the MD, may thus serve as a valuable supplement to enhance the effectiveness of pain management [8,12]. Moreover, our recent research highlighted the pivotal role of the MD that exhibits selective responsiveness to pain processing, distinguishing it from equally salient nonpainful sensory stimuli, such as touch, audition, and vision [13]. Nevertheless, as important as the thalamus is, nociceptive thalamic responses remain inadequately characterized.

Nociceptive stimuli like radiant-heat laser can selectively activate cutaneous nociceptors, primarily Aδ and C fibers, located in the superficial layers of the skin in both humans and animals, thereby eliciting pure painful percepts without tactile sensations [14,15]. The nociceptive information is then conveyed mainly through the spinothalamic tract to the brain [16], ultimately resulting in nociceptive-evoked electrophysiological responses that can be recorded on the scalp surface, or in the epidural space and the cortex [17,18]. These nociceptive-evoked electrophysiological responses encompass event-related potentials (ERPs) like the N1 wave, neural oscillations like gamma-band oscillations (GBOs), and neural spike firing activities, all of which are intricately linked to pain perception [17–20]. However, the majority of previous studies have only recorded nociceptive responses in extracranial and cortical regions [17,20,21]. A systematic characterization of nociceptive-evoked electrophysiological responses in deep brain regions, particularly the thalamus, is still lacking. For example, the N1 wave, which is the early part of cortical nociceptive-evoked ERPs, originates from the S1 and is normally recorded from nearby electrodes [18], but it remains unclear whether an earlier response can also be observed in the thalamus. Several factors contribute to this knowledge gap, with the foremost being the limitations of macro-level electrophysiological techniques like electroencephalography and electrocorticography. These methods suffer from low spatial resolution due to electrode size and placement, as well as the volume conduction effect, rendering them incapable of reliably capturing electrophysiological signals, especially the field potentials, from deep brain regions. Furthermore, the conventional wisdom is that ERPs are predominantly generated by cortical pyramidal neurons, whose vertical alignment enables efficient convergence of postsynaptic potentials [22,23]. Pyramidal neurons in the thalamus, however, do not exhibit the typical organization seen in parallel and vertically aligned cortical structures [24], making it difficult to record ERPs in the thalamus.

**Fig. 1.**
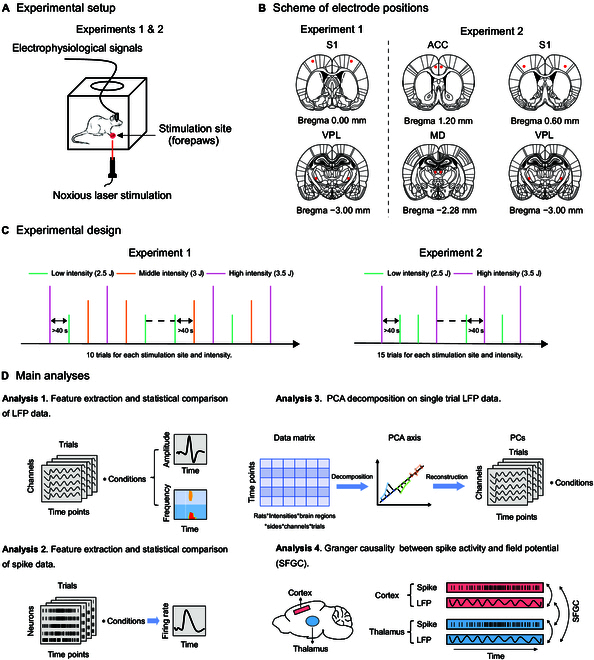
Experimental design and main analyses. (A) The same experimental setup was utilized for both experiments, with rats moving freely within the plastic chamber during recording sessions. Laser stimuli were delivered to the plantar surface of the left or right forepaw when the animals were spontaneously still. (B) Schematic diagram displaying electrode implantation positions for simultaneous recording of multiple brain regions. (C) Experiment 1 involved laser stimulation with 3 intensities (2.5, 3, and 3.5 J), comprising 10 trials for each stimulation site and intensity. Experiment 2 utilized laser stimulation with 2 intensities (2.5 and 3.5 J), with 15 trials for each stimulation site and intensity. All stimuli were pseudo-randomly applied to the rats' forepaws. (D) The study encompassed 4 primary analyses: LFP analysis, spike firing rate analysis, PCA of LFPs, and SFGC analysis.

Given the pivotal role of the thalamus, a complete understanding of pain processing requires a detailed description of how the thalamus responds to nociceptive stimuli. The present study aimed to fill this knowledge gap and provide a systematic characterization of nociceptive thalamic responses. We recorded laser-evoked brain responses (LEPs) in the thalamus (VPL and MD) and cortex (S1 and ACC). We extracted and compared their time-domain features, time-frequency features, and neural spike patterns (Fig. 1). Our investigation revealed that the thalamus exhibited ERP-like responses to laser stimuli (e.g., the N1 wave), and such responses shared many key features with cortical responses. However, the thalamic N1 wave temporally followed its cortical counterpart, contrary to the expected temporal order of ascending information flow. Employing principal component analysis (PCA) and spiking-field Granger causality (SFGC) analysis, we found that the nociceptive thalamic response was well represented by an early negativity (EN) with a latency of around 100 ms. The amplitude of the EN component encoded stimulus intensity and aligned closely in time with the information flow from the thalamus to the cortex. Altogether, these findings unveil the intricate role of the thalamus in pain processing by elucidating a genuine thalamic neural response.

## Results

### Nocifensive behaviors

**Fig. 2.**
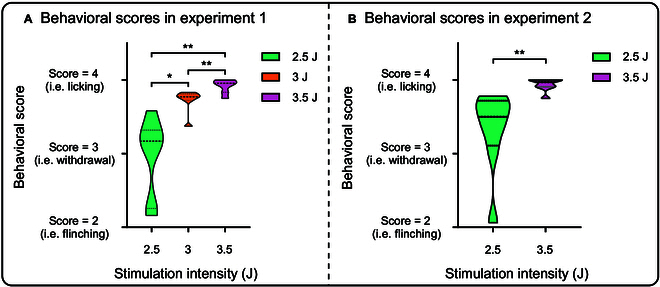
Nocifensive behaviors. (A and B) Nocifensive behavioral scores were significantly influenced by stimulus intensity in both experiments, with notably higher scores observed at greater stimulus intensity. * *P* < 0.05, ** *P* < 0.01.

In 2 experiments (Fig. 1), we delivered multiple laser stimuli of different intensity levels (Experiment 1: 2.5, 3, and 3.5 J; Experiment 2: 2.5 and 3.5 J) to the left and right forepaws of freely moving rats while recording their nocifensive behaviors and electrophysiological responses in the thalamus and cortex (Experiment 1: VPL and S1; Experiment 2: VPL, MD, S1, and ACC). In Experiment 1, we observed a significant modulation of nocifensive behavioral scores based on stimulus intensity (*F*_(2,12)_ = 22.15, *P* = 0.0026, *η_P_^2^* = 0.787), with stronger intensities resulting in higher behavioral scores (mean ± SEM, 2.5 J: 2.96 ± 0.20, 3 J: 3.72 ± 0.06, 3.5 J: 3.91 ± 0.03; 2.5 J vs. 3 J, *P* = 0.0129; 2.5 J vs. 3.5 J, *P* = 0.0054; 3 J vs. 3.5 J, *P* = 0.0057) (Fig. 2A). The behavioral results in Experiment 2 mirrored those in Experiment 1, revealing significantly higher behavioral scores for high-intensity stimuli compared to low-intensity stimuli (mean ± SEM, 2.5 J: 3.32 ± 0.20, 3.5 J: 3.93 ± 0.03; *t*(7) = 3.562, *P* = 0.0092, Cohen’s *d* = 1.259) (Fig. 2B). The behavioral scores for most trials exceeded 3 (i.e., animals retract their paws, lick, or exhibit whole-body movements in response to laser stimulation), indicating that the animals in both experiments experienced pain perception.

### Laser-evoked field potentials

**Fig. 3.**
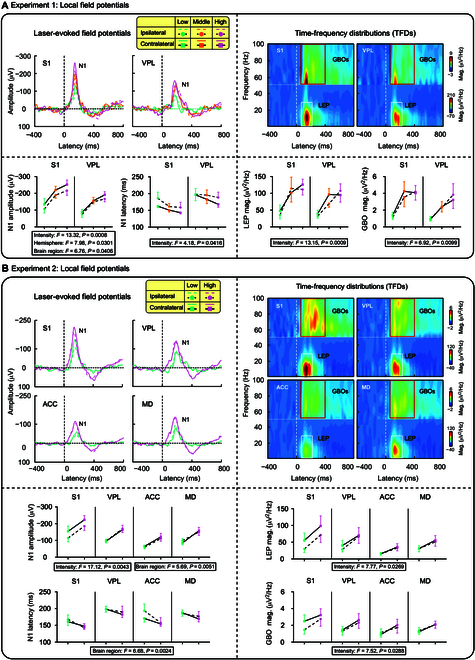
Laser-evoked field potentials. (A) In Experiment 1, N1 amplitudes were significantly influenced by stimulus intensity, hemisphere, and brain region, with no significant interactions. N1 latencies were significantly affected by stimulus intensity. The magnitudes of LEP and GBO were also significantly influenced by stimulus intensity. (B) In Experiment 2, N1 amplitudes were significantly influenced by stimulus intensity and brain region, with no significant interactions. Note that there are significant differences in N1 latencies among brain regions, with significantly shorter latency in S1 than that in VPL. The magnitudes of LEP and GBO were significantly influenced by stimulus intensity. Error bars represent SEM. Grand-averaged waveforms for each condition are presented.

To compare nociceptive-evoked brain responses between the thalamus and cortex, Experiment 1 recorded electrophysiological responses to laser stimuli in the thalamus and cortical brain regions within the lateral pain pathway (i.e., VPL and S1). Experiment 2 aimed to extend the findings of Experiment 1 to more brain regions (i.e., VPL, MD, S1, and ACC), especially considering that the MD and ACC were proven to preferentially encode pain perception [13]. In both experiments, laser stimuli elicited a marked negative wave, traditionally known as the N1 component, in the thalamus and cortex. Along with the time-frequency representation of the low-frequency N1 response (LEP: 80 to 200 ms, 1 to 30 Hz), a prominent brain oscillation in the gamma frequency band (GBO: 70 to 400 ms, 51 to 100 Hz) was observed (Fig. 3A and B). The peak amplitude and latency of the N1, as well as the magnitudes of LEP and GBO, were extracted from each brain region and subsequently analyzed using 3-way (intensity × hemisphere × brain region) repeated-measures analyses of variance (ANOVAs).

In Experiment 1, the thalamus displayed a nociceptive brain response comparable to that of the cortex (Fig. 3A). We observed that N1 amplitudes were significantly affected by the 3 factors (intensity: *F*_(2,12)_ = 13.32, *P* = 0.0008, *η_P_^2^* = 0.689; hemisphere: *F*_(1,6)_ = 7.98, *P* = 0.0301, *η_P_^2^* = 0.570; brain region: *F*_(1,6)_ = 6.76, *P* = 0.0406, *η_P_^2^* = 0.529), with no significant interactions (see Tables S1 and S2 and Fig. 3A). Post hoc pairwise comparisons revealed significantly larger N1 amplitudes to high-intensity stimuli compared to low-intensity stimuli (*P* = 0.0075), with the contralateral hemisphere exhibiting significantly larger N1 amplitudes than the ipsilateral hemisphere (*P* = 0.0301). Additionally, N1 amplitudes in S1 were significantly larger than those in VPL (*P* = 0.0406). N1 latency was solely modulated by stimulus intensity (*F*_(2,12)_ = 4.18, *P* = 0.0416, *η_P_^2^* = 0.411). Middle-intensity stimuli induced a significantly shorter N1 latency compared to low-intensity stimuli (*P* = 0.0308). In the time-frequency domain, the magnitudes of LEP and GBO were also solely significantly influenced by stimulus intensity (LEP magnitudes: *F*_(2,12)_ = 13.15, *P* = 9.439 × 10^−4^, *η_P_^2^* = 0.686; GBO magnitudes: *F*_(2,12)_ = 6.92, *P* = 0.0099, *η_P_^2^* = 0.535). Both middle- and high-intensity stimuli induced larger LEP and GBO magnitudes than low-intensity stimuli (LEP: low vs. middle, *P* = 0.0110, low vs. high, *P* = 0.0190; GBO: low vs. middle, *P* = 0.0471, low vs. high, *P* = 0.0465).

In Experiment 2, N1 amplitudes were strongly modulated by stimulus intensity and brain region (intensity: *F*_(1,7)_ = 17.12, *P* = 0.0043, *η_P_^2^* = 0.709; brain region: *F*_(3,21)_ = 5.69, *P* = 0.0051, *η_P_^2^* = 0.448), with no significant interaction observed (Tables S3 and S4 and Fig. 3B). High-intensity stimuli induced significantly larger N1 amplitudes compared to low-intensity stimuli (*P* = 0.0043), with N1 amplitudes in VPL being significantly larger than those in ACC (*P* = 0.0224). Moreover, significant differences in N1 latency were found between brain regions (*F*_(3,21)_ = 6.68, *P* = 0.0024, *η_P_^2^* = 0.488). The N1 latency in S1 was significantly shorter than that in VPL (*P* = 0.0138). In the time-frequency domain, both LEP and GBO magnitudes were significantly modulated by stimulus intensity (LEP magnitudes: *F*_(1,7)_ = 7.77, *P* = 0.0269, *η_P_^2^* = 0.526; GBO magnitudes: *F*_(1,7)_ = 7.52, *P* = 0.0288, *η_P_^2^* = 0.517). LEP and GBO magnitudes induced by high-intensity stimuli were significantly larger than those induced by low-intensity stimuli.

In summary, both experiments highlighted a notable impact of stimulus intensity on laser-evoked field potentials in both the thalamus and cortex. Notably, Experiment 2 unveiled a significant difference in N1 latency between the cortex and thalamus, with VPL displaying a longer N1 latency compared to S1. In Experiment 1, the N1 latency in VPL was also on average longer than that in S1. These observations suggested that nociceptive thalamic responses paradoxically occurred later than those measured from the S1.

### Laser-induced spike activities

**Fig. 4.**
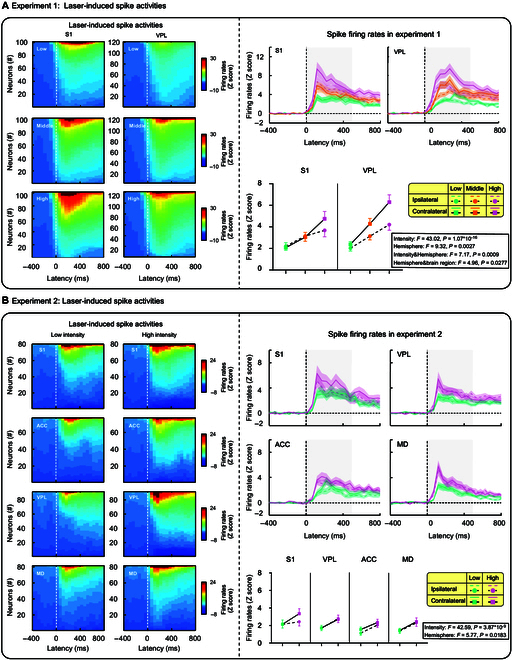
Laser-induced spike activities. The firing rates of single units were normalized as Z-scores relative to the baseline (500 ms preceding laser stimuli). Units were arranged on the y-axis to visualize the relative changes in firing rates (left panel of [A] and [B]). In both experiments, spike firing rates were represented as the average curve with an error band (standard error), and the firing rates (gray shaded area) within the 0- to 500-ms time window post-stimulation were used for statistical analysis. (A) In Experiment 1, spike-firing rates increased with stimulus intensity in both hemispheres and brain regions. Notably, the firing rates in contralateral VPL were significantly higher than those in ipsilateral VPL. (B) In Experiment 2, spike-firing rates in the 4 brain regions were significantly affected by intensity and hemisphere. Error bars represent SEM. Grand-averaged spike firing waveforms for each condition are presented.

To compare neuronal firing patterns between the thalamus and cortex, we extracted single-unit spike activities from the laser-evoked electrophysiological responses. In the 2 experiments, a total of 551 units exhibiting discernible spike responses were identified (Experiment 1: 222 units, [S1] 101 units, [VPL] 121 units; Experiment 2: 329 units, [S1] 80 units, [VPL] 91 units, [ACC] 77 units, [MD] 81 units). A summary of the spike firing activities evoked by nociceptive stimuli was provided in Fig. 4 and Table S5.

In Experiment 1, we found that spike-firing rates were influenced by both intensity and hemisphere factors, with 2 significant interactions (intensity: *F*_(2,240)_ = 43.02, *P* = 1.07 × 10^−16^, *η_P_^2^* = 0.263; hemisphere: *F*_(1,120)_ = 9.32, *P* = 0.0027, *η_P_^2^* = 0.072; intensity × hemisphere interaction: *F*_(2,240)_ = 7.17, *P* = 0.0009, *η_P_^2^* = 0.056; hemisphere × brain region interaction: *F*_(1,120)_ = 4.96, *P* = 0.0277, *η_P_^2^* = 0.039). Post hoc pairwise comparisons indicated that higher stimulus intensity led to larger spike-firing rates in both brain hemispheres (all *P* < 0.05). Being evoked by high-intensity stimuli, spike-firing rates were significantly larger in the contralateral hemisphere than in the ipsilateral hemisphere (*P* = 1.555 × 10^−4^). This hemisphere effect was more evidently observed in the VPL (*P* = 3.087 × 10^−6^). Additionally, the firing rates in contralateral VPL were significantly higher than those in contralateral S1 (*P* = 0.0396).

In Experiment 2, we found that spike-firing rates were also influenced by both intensity and hemisphere factors (intensity: *F*_(1,90)_ = 42.59, *P* = 3.981 × 10^−9^, *η_P_^2^* = 0.321; hemisphere: *F*_(1,90)_ = 5.77, *P* = 0.0183, *η_P_^2^* = 0.061). Spike-firing rates evoked by high-intensity stimuli were significantly larger than those evoked by low-intensity stimuli in all brain regions. Furthermore, the contralateral hemisphere exhibited significantly larger spike-firing rates than the ipsilateral hemisphere. Taken together, these results indicated that stimulus intensity modulated the spike-firing activities of all thalamic and cortical brain regions, which were stronger in the brain hemisphere contralateral than ipsilateral to nociceptive stimuli. This observation aligns with well established anatomical principles of the spinothalamic tract [7] and is supported by previous findings in electrophysiological studies [18,21].

### Isolation of EN in thalamic field potentials

**Fig. 5.**
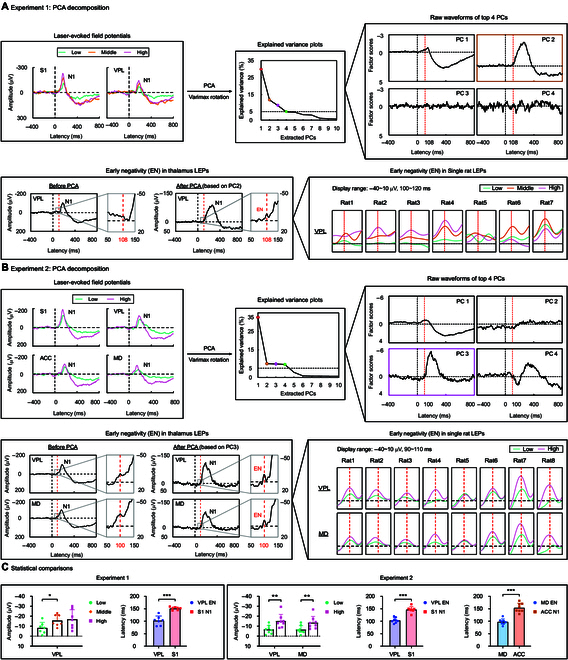
PCA to isolate the EN in the thalamus. (A and B) In both experiments, 4 principal components were extracted, with their explained variance shown in the eigenvalue graph (Experiment 1: [PC1] 30.58%, [PC2] 11.87%, [PC3] 8.56%, [PC4] 4.82%; Experiment 2: [PC1] 32.57%, [PC2] 7.49%, [PC3] 7.33%, [PC4] 7.03%). PC2 in Experiment 1 and PC3 in Experiment 2 closely resembled the original N1 wave but revealed a distinct EN at 108 and 100 ms, respectively. Reconstructed field potentials based on PC2 in Experiment 1 and PC3 in Experiment 2 consistently demonstrated the presence of EN in all rats. (C) In Experiment 1, the modulatory effect of stimulus intensity on thalamic EN amplitudes was significant, with the EN amplitudes elicited by middle-intensity stimuli being significantly higher than those induced by low-intensity stimuli. In Experiment 2, EN amplitudes were also significantly influenced by stimulus intensity. EN amplitudes elicited by high-intensity stimuli in both thalamic regions were significantly larger than those induced by low-intensity stimuli. Furthermore, in both experiments, thalamic EN latencies were significantly shorter than cortical N1 latencies in both pain pathways. * *P* < 0.05, ** *P* < 0.01, *** *P* < 0.001. Error bars represent SEM. Grand-averaged waveforms of the field potentials and single rat waveforms of the EN component are presented.

Local field potential (LFP) results suggested that the N1 wave in the S1 preceded that in the thalamus. This finding was unexpected and seemed to contradict the functional projections from the thalamus to the cortex [25]. A reasonable explanation for this contradiction would be that the thalamic N1 is not generated in the thalamus per se but is a product of volume conduction or feedback projection signals from the cortex. To explore whether there is any genuine nociceptive thalamic response, we then employed a hypothesis-free approach using PCA decomposition with Varimax rotation to separate distinct noxious responses (Fig. 5A and B). In both experiments, 4 principal components (PCs) accounted for more than or about 5% of the signal variance: Experiment 1, PC1 (30.58%), PC2 (11.87%), PC3 (8.56%), PC4 (4.82%); Experiment 2, PC1 (32.57%), PC2 (7.49%), PC3 (7.33%), PC4 (7.03%). Notably, in both experiments, a PC (PC2 in Experiment 1 and PC3 in Experiment 2) exhibited waveform characteristics highly similar to the N1 wave (Experiment 1: Pearson *r* = 0.72, *P* < 0.001; Experiment 2: Pearson *r* = 0.74, *P* < 0.001) but featured with an early negative response of a smaller amplitude at approximately 100 ms.

To further elucidate the contribution of each PC on the original signals in each brain region, we inverse-transformed each PC separately back to the original feature space and then subtracted these reconstructed signals from the original signals. The results showed that PC1 in both experiments significantly influenced the N1 wave and the subsequent positive deflection. In contrast, PC2 in Experiment 1 and PC3 in Experiment 2 primarily affected the N1 and EN components in the thalamus (Fig. S2). Notably, the presence of EN became more evident in PC2 in Experiment 1 and PC3 in Experiment 2. Subsequently, we reconstructed the EN-related PCs at the single-subject level to evaluate the variation of EN and found that EN was present in every single rat (Fig. 5A and B). To assess the reliability of EN components, we also conducted PCA exclusively using cortical data or thalamic data. Cortical data alone were unable to extract PCs with the EN-like component, whereas single thalamic data can (Fig. S3). More importantly, we performed the same PCA using tactile-evoked potentials also collected in Experiment 2 [13] but did not observe any response similar to the EN component isolated from laser-evoked potentials (Fig. S4). These findings further support the notion that the EN would be a genuine nociceptive thalamic response.

To investigate whether the EN could carry nociceptive information, we examined its amplitudes under different stimulus intensity conditions (Fig. 5C). In Experiment 1, a significant regulatory effect of stimulus intensity on the EN amplitudes was observed (*F*_(2,12)_ = 4.86, *P* = 0.0284, *η_P_^2^* = 0.447), with the EN amplitudes elicited by middle-intensity stimuli being significantly higher than those induced by low-intensity stimuli (*P* = 0.0244). In Experiment 2, EN amplitudes were also significantly influenced by stimulus intensity (intensity: *F*_(1,7)_ = 20.75, *P* = 0.0026, *η_P_^2^* = 0.747). Post hoc pairwise comparisons demonstrated that EN amplitudes elicited by high-intensity stimuli in both thalamic regions were significantly larger than those elicited by low-intensity stimuli (VPL, *P* = 0.0031; MD, *P* = 0.0024). Additionally, we extracted and compared the latencies of the thalamic EN and the cortical N1, revealing that EN preceded the cortical N1 in both experiments (Experiment 1: [EN in VPL vs. N1 in S1] *t*(6) = 7.100, *P* = 0.0004, Cohen’s *d* = 2.683; Experiment 2: [EN in VPL vs. N1 in S1] *t*(7) = 7.232, *P* = 0.0002, Cohen’s *d* = 2.556, [EN in MD vs. N1 in ACC] *t*(7) = 6.218, *P* = 0.0004, Cohen’s *d* = 2.198; Fig. 5A and B).

### Granger causality between spikes and LFPs in the thalamus and cortex

**Fig. 6.**
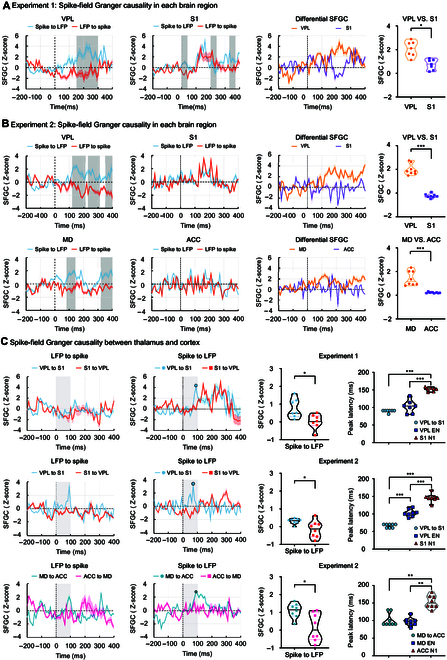
SFGC of brain responses in the thalamus and cortex. The SFGC time courses for both experiments are presented as average curves with standard error bands. (A and B) Dark gray shadows highlight significant time intervals, determined through point-by-point paired-sample *t* tests for information flow within each brain region. In Experiment 1, VPL exhibited significantly higher differential information flow (i.e., Spike-to-LFP minus LFP-to-Spike) than S1. Experiment 2 also showed higher differential information flow within the thalamus (i.e., VPL and MD) compared to the cortex (i.e., S1 and ACC). (C) In both experiments, information flow from the thalamus to the cortex peaked before 100 ms. The information flow from the thalamic spikes to the cortical LFPs at 0 to 100 ms was significantly stronger than that from the cortex to the thalamus. Moreover, significant differences in peak latency were observed between (1) the information flow from the thalamus to the cortex, (2) the EN in the thalamus, and (3) the N1 in the cortex. In Experiment 1, the peak latencies of the information flow from VPL to S1 and the EN in VPL occurred significantly earlier than the N1 in S1. In Experiment 2, the peak latency of the information flow from VPL to S1 was significantly earlier than the peak latencies of the EN in VPL and the N1 in S1. The peak latency of the EN in VPL was also significantly earlier than that of the N1 in S1. Furthermore, the peak latencies of the information flow from MD to ACC and the EN in MD also occurred significantly earlier than the peak latency of the N1 in ACC. * *P* < 0.05, ** *P* < 0.001, *** *P* < 0.001. Grand-averaged SFGC waveforms for each condition are presented.

To explore the causal relationship of brain responses within the same brain regions or between different brain regions, we calculated the SFGC to estimate the directional interaction between discrete spike signals and continuous field potential signals in 2 experiments. Our analyses revealed distinct information flow within and between the thalamus and cortex (Fig. 6).

We conducted point-by-point paired-sample *t* tests on the SFGC information flow within each brain region, corrected by false discovery rates, to identify time intervals with significant differences in information flow in different directions. We found a marked difference in information flow within the thalamus (Fig. 6A and B). Specifically, we observed a significant disparity in differential information flow (spike-to-LFP SFGC minus LFP-to-spike SFGC) between the thalamus and cortex within the 0 to 400 ms following stimulation. In Experiment 1, the comparison of differential information flow showed significant differences between VPL and S1 (*t*(6) = 3.48, *P* = 0.013, Cohen’s *d* = 1.318). In Experiment 2, the differential information flow between VPL and S1 (*t*(7) = 14.25, *P* = 19.905 × 10^−7^, Cohen’s *d* = 5.038) and between MD and ACC (*t*(7) = 5.57, *P* = 0.0008, Cohen’s *d* = 1.971) were also significant. Interestingly, we observed that the peak latency of the spike-to-LFP SFGC from the thalamus to cortex occurred before 100 ms (Fig. 6C). Moreover, the SFGC from the thalamus to the cortex during the 0- to 100-ms interval was significantly stronger than that from the cortex to the thalamus (Experiment 1: [VPL-to-S1 vs. S1-to-VPL] *t*(6) = 2.570, *P* = 0.0423, Cohen’s *d* = 0.971; Experiment 2: [VPL-to-S1 vs. S1-to-VPL] *t*(7) = 2.421, *P* = 0.0460, Cohen’s *d* = 0.856; [MD-to-ACC vs. ACC-to-MD] *t*(7) = 2.800, *P* = 0.0265, Cohen’s *d* = 0.989).

Furthermore, in both experiments, significant differences were observed between the peak latency of the SFGC from thalamic spikes to cortical LFP and the peak latencies of the thalamic EN and cortical N1 components. In Experiment 1, there were significant differences in these 3 latencies (*F*_(2,6)_ = 53.16, *P* = 0.0001, *η_P_^2^* = 0.898). Post hoc comparisons indicated that the peak latency of the information flow from VPL to S1 and the peak latency of the EN component in VPL were significantly earlier than that of the N1 component in S1 (EN of VPL vs. N1 of S1: *P* = 0.0009; VPL-to-S1 SFGC vs. N1 of S1: *P* = 10^−6^). In Experiment 2, significant latency differences were also observed in both lateral and medial pain pathways ([lateral] *F*_(2,7)_ = 140.79, *P* = 8.20 × 10^−8^, *η_P_^2^* = 0.952; [medial] *F*_(2,7)_ = 23.2, *P* = 0.0005, *η_P_^2^* = 0.768). Post hoc comparisons revealed that the peak latency of the information flow from VPL to S1 was significantly earlier than the peak latencies of the EN in VPL and the N1 in S1, and the peak latency of the EN in VPL was also significantly earlier than that of the N1 in S1 (EN of VPL vs. N1 of S1: *P* = 0.0004; VPL-to-S1 SFGC vs. N1 of S1: *P* = 9.20 × 10^−7^; VPL-to-S1 SFGC vs. EN of VPL: *P* = 4.52 × 10^−5^). Moreover, the peak latency of the information flow from MD to ACC and the peak latency of the EN in MD were also significantly earlier than that of the N1 in ACC (EN of MD vs. N1 of ACC: *P* = 0.0011; MD-to-ACC SFGC vs. N1 of ACC: *P* = 0.0077). These results suggest that thalamic spikes exert a directional impact on cortical field potentials, with a significantly shorter latency than the N1 component of the cortex. However, there was no consistent latency difference between the thalamus-to-cortex information flow and the EN component of the thalamus, supporting the speculation that the EN component is a genuine neural response of the thalamus.

## Discussion

Despite the key roles of the thalamus in both the lateral and medial pain pathways, nociceptive thalamic responses are rarely recorded and characterized in pain electrophysiology studies. Here, to extract genuine nociceptive thalamic responses, we used a rat pain model with the radiant-heat laser to elicit pure pain experience, which has previously been used to study nociceptive-evoked cortical responses [18,26–29]. By simultaneously recording laser-evoked neural responses in the thalamus and cortex in 2 experiments (Fig. 1B), we obtained 3 key findings. First, while the thalamus exhibited nociceptive-evoked neural responses similar to the cortex (Figs. 3 and 4), the recorded thalamic responses were temporally later than their cortical counterparts, which is contrary to the expected temporal sequence of ascending information flow. This observation suggested that these responses may not be originated from the thalamus (Fig. 3). Second, PCA revealed a distinctive EN component at approximately 100 ms, with amplitude grading linked to stimulus intensity and a peak latency shorter than subsequent N1 wave (Fig. 5). This result suggested that the EN component may represent an authentic nociceptive thalamic response. Third, Granger causality (GC) analysis between the thalamus and cortex provided compelling evidence that thalamic spikes exerted a directional influence on cortical LFP signals at around 100 ms, aligning with EN latency and preceding cortical N1 wave (Fig. 6). This finding further suggested that the EN component would be a genuine thalamic response. Taken together, these findings demonstrate that although the thalamus shows typical nociceptive responses as the cortex, it has its distinct and genuine nociceptive responses (i.e., EN), which would be important to be investigated for a comprehensive understanding of the thalamic function in pain processing.

With a well-validated animal pain model [19,30,31], we systematically characterized thalamic responses to nociceptive laser stimuli. We found that typical laser-evoked brain responses, like the N1 wave, GBOs, and spike firing patterns, could be recorded in the thalamus (VPL and MD) and the cortex (S1 and ACC) alike. These responses were all responsive to different intensity levels of nociceptive stimuli: larger responses were consistently elicited by high-intensity stimuli than low-intensity stimuli. Notably, prior studies in humans have established a robust association between scalp-recorded LEPs and the perception of pain intensity [17,20,32] and provided evidence that LEPs can serve as a reliable and selective indicator of an individual’s ability to discriminate between different levels of pain [23]. The present study corroborates these conclusions by showing that laser-evoked brain responses in S1 and ACC, which are among the neural origins of scalp-recorded LEPs, can encode information about pain intensity. Interestingly, we found that across brain regions, the GBOs could stably encode nociceptive stimulus intensity. This finding aligns with abundant prior research showing that GBOs in S1 track both within- and between-individual pain sensitivity across species independent of stimulus saliency [17,19,33–35] and further support GBO’s role as a reliable indicator of pain intensity. Although our findings confirm that the thalamus and the cortex share many features and functions in their responses to nociceptive stimuli, it is crucial to recognize that these typical brain responses (e.g., the N1 wave collected from the thalamus) may not represent an authentic thalamic nociceptive response, cautioning against directly interpreting it as a “thalamic” nociceptive response.

While the typical brain responses were not generated from the thalamus, the thalamus still has its distinct nociceptive-evoked responses. Specifically, different from the N1 wave that originated from the cortex, the EN seems to be a unique nociceptive response that the thalamus generates. Three key pieces of evidence support this statement. First, the thalamic N1 wave consistently lagged the cortical N1 wave in both lateral and medial pain pathways (Tables S1 and S3). This observation is incompatible with the temporal order of ascending transmission of nociceptive information from the thalamus to the cortex. Second, PCA successfully extracted a thalamus-weighted PC in both experiments, consistently revealing an EN component around 100 ms after stimulus onset in the reconstructed LEP waveforms (Fig. 5A and B). The EN amplitude was influenced by stimulus intensity, with higher-intensity stimuli inducing larger EN amplitudes. This finding moves beyond past research by directly illustrating the role of thalamic LFP responses in encoding pain intensity, surpassing the prior emphasis on thalamic spike activity in pain pathways [36]. Furthermore, the peak latency of the thalamic EN was significantly earlier than the cortical N1 latency (Fig. 5A and B), suggesting that the EN could be an authentic thalamic response to nociceptive stimuli. Finally, GC analysis revealed evident spike-to-LFP information flow from the thalamus to the cortex, with a peak latency before 100 ms. Importantly, this thalamic-cortical information flow occurred significantly earlier than the N1 component in the cortex, while its peak latency closely matched the EN component of the thalamus (Fig. 6C). Notably, our study identified the EN component both in the MD and VPL, indicating that EN may be a universal thalamic feature, rather than limited to a specific thalamic nucleus.

What is the N1 component observed in the thalamus if it is not a thalamus-generated response? Plausible speculations would be that it is (a) a result of the volume conduction of the cortical N1 signal or (b) the feedback signal from the cortex to the thalamus. Notably, there exists a close anatomical projection and functional connection between the thalamus and cortex. The thalamus provides feedforward input to the cortex, particularly in the fourth layer of the cortex, while the cortex sends feedback to the thalamus through neurons in the fifth and sixth layers [37]. It is thus physiologically plausible that the cortex sends feedback signals to the thalamus. However, this speculation is not likely to be true. The thalamus lacks the regular, vertically arranged neuronal structure, which is often seen in the cortex and is key to the generation of evoked potentials [38]. Thalamic neurons are organized in a relatively disordered manner, which limits the convergence of postsynaptic potentials and is unlikely to generate large evoked potentials like the N1 wave. Additionally, substantial differences exist in the morphological and electrophysiological properties of thalamic and cortical neurons. Thalamic relay neurons, characterized by a dendritic structure, primarily project to the cortex and other brain regions, displaying a distinctive tonic and burst discharge pattern absent in cortical neurons [24]. These variations in anatomical connections and neuronal properties likely account for the absence of the N1 wave in the thalamus.

Mechanically, our findings are supported by a recent study utilizing advanced data analyses and neural circuit intervention techniques [26]. This work indicates that the initial waves in thalamic field potentials align with the local origin of peripheral inputs, while the cortex exerts a notable influence in subsequent time windows of thalamic field potentials [26]. Additionally, previous studies have elucidated the bidirectional control of cortical activities on thalamic nociceptive responses [37,39]. In particular, microinjection of gamma-aminobutyric acid antagonists on corticofugal neurons enhanced the nociceptive-evoked response of VPL neurons [40]. Furthermore, the activation of cortical output neurons in layer 6 (L6) enhanced thalamic somatosensory responses and drove aversive hypersensitivity and spontaneous nocifensive behavior [39]. These previous findings underscore the intricate and pivotal bidirectional control role of the cortex in thalamic nociceptive responses, thus offering mechanistic support for our current research and suggesting that the early EN component identified in our study represents an authentic nociceptive thalamic response. Future research could explore whether analogous mechanisms underlie thalamic nociceptive responses in both humans and animals, contributing to translational applications.

Considering that field potential signals can offer information distinct from spikes and maintain a reliable signal-to-noise ratio during chronic recordings [41], this study holds both theoretical significance and clinical implications. Theoretically, our research uncovers both shared and distinctive electrophysiological responses between the thalamus and cortex, deciphering a genuine thalamic neural response, i.e., the EN component. The current study not only fills a knowledge gap in thalamic electrophysiology but also emphasizes the divergent electrophysiological profiles and functional roles of the thalamus in pain processing. Given the pivotal role of the thalamus in the central nervous system and its regulatory function in global information processing [42], the thalamic nucleus is often considered an effective target for the treatment of neurological diseases [43,44]. Clinically, the identification of genuine thalamic neural responses opens avenues for its application as an objective symptom assessment and for therapeutic monitoring in closed-loop neuromodulation based on thalamic targets. Specifically, as an authentic thalamic response capable of encoding pain intensity, EN offers potential neural indicators that reflect pain perception and relief. This capability facilitates the dynamic adjustment of neuromodulation parameters, enabling adaptive and personalized pain management interventions. The incorporation of EN into closed-loop systems may enhance precision and responsiveness, hence optimizing the effectiveness of neuromodulation treatments for individuals with chronic pain.

Several noteworthy limitations should be acknowledged. First, this study was unable to isolate specific neural responses in the lateral and medial pain pathways. Future research may use tailored stimuli, drug interventions targeting pathway-specific neurotransmitters, and genetic manipulation (optogenetics or chemogenetics) to address this challenge. Due to the inherent link between sensory and emotional aspects of pain, fully isolating these components remains a formidable task. Second, the reliance solely on electrophysiological techniques in the current experimental design may constrain the prospect of investigating the neural origin and functional importance of the EN component. Future research incorporating advanced methods like microdialysis and genetic manipulation is needed to address this issue. Third, future studies should include data from female animals to ensure a more balanced and comprehensive perspective on our findings. This consideration is crucial given the recognized importance of gender differences in animal pain research.

## Materials and Methods

### Animals

A total of 20 adult male Sprague Dawley rats, weighing between 350 and 450 g, were utilized in 2 experiments. Five rats were excluded due to electrode misalignment, breakage, or abnormal signal, resulting in the inclusion of 7 rats in Experiment 1 (excluded *n* = 3, 2 for electrode misalignment and 1 for electrode breakage) and 8 rats in Experiment 2 (excluded *n* = 2, 1 for electrode misalignment and 1 for field potentials signal abnormality). The rats were individually housed in different cages maintained at controlled temperature and humidity conditions, with 1 rat per cage (cage size: 330 × 250 × 220 mm^3^). They were maintained on a 12-h light/dark cycle and access to food and water ad libitum. Experimental animal selection adheres to the 3Rs principle, optimizing reliability while minimizing animal use. To maintain consistency with prior research and eliminate menstrual period interference with pain perception, only male animals were employed [27,45,46]. All surgical and experimental procedures adhered to the guidelines of the Committee for Research and Ethical Issues of the International Association for the Study of Pain [47]. These procedures received approval from the Animal Care and Use Committee of the Institute of Psychology, Chinese Academy of Sciences (Approval number: H22033).

### Surgical procedures and electrode implantation

Detailed surgical procedures are documented in our previous publications [27,30]. Prior to the surgery, rats were anesthetized with isoflurane at a concentration of 5% (v/v) with an airflow rate of 1 l/min in an induction chamber (RWD Life Science, China). During the surgery, the rat's head was secured using a stereotaxic apparatus and maintained under anesthesia via an anesthetic mask at a concentration of 2% (v/v) with an airflow rate of 0.5 l/min. Depth of anesthesia during surgery was assessed by regularly testing the hindpaw withdrawal and tail-pinch reflexes. For multiregion electrophysiological recording, several groups of tungsten microfilament electrodes (20 μm in diameter, California Fine Wires Company, USA) were surgically implanted into rat brain tissues. Tetrode electrodes (impendence: 2 to 2.5 MΩ) were adopted to enhance the detection of single-unit spike activity. Before surgical implantation, these electrodes were immersed in DiI solution (cell red fluorescent dye, Yeasen Biotechnology, China) to mark the location of the electrode tips (Fig. S1).

All electrodes were assembled within a 3-dimensional (3D)-printed module and safeguarded by a 3D-printed shell. Without penetrating the underlying dura mater, 2 stainless steel screws (diameter: 1 mm) were positioned as reference and ground electrodes, 2 and 4 mm caudally to the lambda, respectively. The implantation coordinates of each brain region were determined following the sixth edition of rat brain atlas [48], and all electrodes were bilaterally implanted in the hemispheres (Fig. 1B). In Experiment 1, electrode implantation coordinates were as follows: S1 (AP: +0 mm, ML: ±3.9 mm, depth: −0.5 mm) and VPL (AP: −3.0 mm, ML: ±3.3 mm, depth: −6.0 mm). In Experiment 2, S1 coordinates were slightly modified to ensure unobstructed placement of the 3D protective base at the craniotomy site's external position. The electrode implantation coordinates for each brain region were as follows: S1 (AP: +0.6 mm, ML: ±3.2 mm, depth: −1.5 mm), VPL (AP: -−3.0 mm, ML: ±3.0 mm, depth: −6.0 mm), ACC (AP: +1.2 mm, ML: ±0.6 mm, depth: −1.2 mm), and MD (AP: −2.3 mm, ML: ±0.8 mm, depth: −5.0 mm). The depth coordinate indicates the distance from the surface of the brain to the targeted brain region. To prevent postsurgical infections and facilitate recovery, all rats received a penicillin injection (800,000 U, i.p.) and rodent nutritional supplements 3 d after the surgery. Rats were individually housed for a minimum of 7 d before any experimental procedures, and their recovery state was assessed by monitoring fur color, mouth and nose color, and body weight.

### Animal sacrifice and brain histological examination

At the end of the experiment, rats were sacrificed by an excessive amount of isoflurane and subsequently perfused with phosphate-buffered saline followed by 4% paraformaldehyde (PFA) through the heart. Brains were extracted, soaked in PFA, postfixed in PFA overnight at 4°C, and then transferred to a 30% sucrose solution until saturated (for 36 to 48 h). Subsequently, the brains were embedded in an optimal cutting temperature embedding agent, and coronal brain slices (40 μm) were cut using a freezing microtome (CM3050S, Leica Biosystems Nussloch GmbH, Germany). Digital microscopy imaging was employed to capture images of the brain tissue slices, confirming the electrode implantation site through DiI expression (DMI8, Leica Microsystems IR GmbH, Germany).

### Experimental protocol

Transient radiant-heat stimuli were generated by an infrared neodymium yttrium aluminum perovskite (Nd: YAP) laser with a wavelength of 1.34 mm (Electronical Engineering, Italy). This nociceptive stimulation can directly activate the nociceptive fibers in the superficial skin layers without co-activating tactile fibers [49–51]. The laser beam was transmitted to the stimulated site via optical fiber, and its spot diameter was set at approximately 5 mm (equivalent to about 20 mm^2^) by focusing lens. Each laser pulse had a duration of 4 ms. Laser pulses were directed at the hairless skin on both the left and right forepaws of the rats under He-Ne laser irradiation. In Experiment 1, stimulus energies of 2.5, 3, and 3.5 J were used, while Experiment 2 employed stimulus energies of 2.5 and 3.5 J. To prevent sensitization and fatigue of nociceptors, the laser focus was shifted after each stimulus [27]. In both experiments, a consistent interval of over 40 s was maintained between 2 consecutive stimuli [18] (Fig. 1C). Before recording neural responses elicited by laser stimuli, in Experiment 2, tactile-evoked brain responses were also collected, with an inter-block interval of at least 30 min. The electrical stimulus comprised square wave impulses lasting 1 ms, delivered at both low (500 μA) and high (1 mA) intensities, respectively. Tactile stimulation was applied in the same way as pain stimulation, randomly to the hairless skin of the left and right forepaws of rats at specified intervals (>20s).

Prior to data collection, rats were acclimated to the recording setting environment by placing them in a plastic chamber for 1 h. During the data collection phase, rats were permitted to move freely within the chamber. Laser stimuli were administered in an alternative manner to either the left or right forepaw's plantar surface through openings in the chamber floor when the animal was spontaneously motionless. In Experiment 1, 10 pulses were randomly administered for each stimulus intensity and each stimulus site, and in Experiment 2, 15 pulses were delivered for each stimulus intensity and each stimulus site. The total number of pulses administered in both experiments amounted to 60 (Fig. 1C). This adjustment of intensity levels and trial numbers in Experiment 2 aimed to enhance the signal-to-noise ratio, streamline the experimental design, and simplify operational procedures. To record tactile-evoked brain responses, Experiment 2 delivered 20 electrical stimuli, which were randomly applied at each intensity and stimulus site, resulting in 80 trials in total. To capture nociceptive-selective brain responses while minimizing any laser-generated ultrasound-related auditory brain responses, white noise at a level of 70 dB was continuously played during data collection [25]. Nocifensive behaviors exhibited by the rats in response to laser stimuli throughout the experiment were assessed using established criteria previously defined and employed [19,29,52]. The specific scoring criteria are as follows: no movement (score = 0), head turning (including shaking or elevating the head; score = 1), flinching (i.e., a small abrupt body jerking movement; score = 2), withdrawal (i.e., paw retraction from the laser stimulus; score = 3), licking, and whole-body movement (score = 4).

### Electrophysiological data collection and data analysis

Electrophysiological signals were recorded with a sampling rate of 40 kHz using the Plexon system amplifier, and the electrode interface board was connected to the 32-channel headstage (Plexon Inc, USA). Subsequently, the raw wide-band data were preprocessed using NDManager [30] and further analyzed through EEGLAB [53] in combination with custom-written MATLAB scripts. Only high-quality contacts were included in the electrophysiological data analysis, and at least 1 good channel was preserved in each brain region. If multiple channels were retained, their field potential signals would be averaged, as they belong to the same brain region and exhibit comparable signal characteristics.

#### Time–domain and time-frequency analyses

The LFP signals were resampled to 1000 Hz and band-pass filtered between 1 and 100 Hz to remove low-frequency fluctuations and high-frequency noises. Furthermore, a notch filter between 48 and 52 Hz was applied to remove 50-Hz power line artifacts. Data epochs were extracted from the continuous recordings using a time window of 1500 ms (consisting of 500-ms prestimulus and 1000-ms post-stimulus intervals). These epochs were baseline-corrected by subtracting the signals within the prestimulus interval. The bad channels and artifacts contaminated trials were excluded by visual inspection. Following the methodology used in previous studies [18,29], the peak amplitude and latency of the N1 wave were extracted from subject-level average waveforms within the 100 to 300 ms post-stimulus. To obtain the group-level LEP waveforms, the subject-level averaged waveforms were subsequently averaged across subjects based on the experimental conditions (Fig. 1D). Time-frequency distributions of laser-evoked responses were calculated using a windowed Fourier transform with a fixed 100-ms Hanning window. For each epoch, a complex time–frequency spectrum estimate F(t, f) was generated across a predefined time range (−500 to 1000 ms in 1-ms increments) and frequency range (1 to 100 Hz in 1-Hz steps). The spectrogram, P(t, f) = |F(t, f)|^2^, represents the power spectral density as a joint function of both time and frequency at each time-frequency point (Fig. 1D). To avoid the bias caused by the windowing and padding operations, a shorter prestimulus interval (i.e., −400 to −100 ms relative to stimulus onset) was selected for baseline correction with the subtraction method [54]. The magnitudes of time-frequency features were extracted by averaging the spectral power within predefined time-frequency windows of interest: LEP, 80 to 200 ms, 1 to 30 Hz; GBO, 70 to 400 ms, 51 to 100 Hz.

#### Principle component analysis

To isolate potential authentic thalamic responses, a PCA decomposition with Varimax rotation was conducted in the time domain [55]. The PCA decomposition was applied to single-trial epochs of filtered LFP data. Specifically, single-trial epochs from all electrodes, all experimental conditions, and all rats were organized as vectors and stacked to create a 2-dimensional data matrix (i.e., time points × trials). This matrix was then decomposed into a set of PCs. The Varimax rotation algorithm was employed to maximize the sum of the variances of the squared loadings, thereby providing an optimal description of the matrix using a linear combination of several basis functions [55] (Fig. 1D). The 4 PCs with the highest variance were selected, with each exhibiting an explanation percentage of variance greater than or close to 5% [19,56]. These PCs were then projected back into the original feature space to reconstruct signals for each PC. In addition, we subtracted the reconstructed data for each PC from the original signals to identify a PC with a great impact on the recorded neural signals in both experiments (Fig. S2).

#### Spike detection

For retained individual good channels, we also conducted spike sorting using KlustaKwik (http://klustakwik.sourceforge.net/). To detect the spiking activity of single units, we employed the standard fixed threshold method, setting the amplitude threshold at 3 standard deviations of the noise floor. This enabled us to extract signals containing multiunit spikes from electrophysiological data filtered with a high-pass filter at 300 Hz. Subsequently, manual adjustments and verifications were performed using the Klusters software [30]. The sorted single-unit data were segmented into a 1500-ms time window (−500 to 1000 ms with respect to stimulus onset), and the spike density was calculated to characterize the spike firing activity. The firing rates of single trials were binned using a 50-ms time window and normalized to the baseline using the *z*-score method [18], which involved subtracting the prestimulus mean values from each data point and then dividing the result by the standard deviation measured during the prestimulus interval. Units with extremely low firing rates, specifically less than 1 Hz during the post-stimulus 1000-ms period, were excluded from further analyses.

#### Spike-field Granger causality analysis

To evaluate the relationship between spiking firing and field potentials, the SFGC was calculated using a model-free, nonparametric method [57]. This approach directly employs the "binless" spikes and the spectral estimate of spike time sequences rather than spike counts. Specifically, the power spectra for the discrete spiking point process *S_x_( f )* and continuous LFP time series *S_y_( f )* were both estimated with the multitaper method [58]. Then, the cross-spectrum *S_xy_( f* ) between LFP and spike train was calculated. The cross-spectrum matrix *S_xy_( f )* was then factorized using Wilson’s algorithm [59,60] for the estimation of SFGC [61,62]. To capture the time-varying characteristics of GC more effectively, spectral information was integrated within the frequency range of 0 to 100 Hz. Additionally, baseline correction was applied to the time course of GC using the *z*-score method. This analysis enabled us to explore the causal relationship between spikes and LFP signals, providing insights into the temporal dynamics of how thalamic/cortical spikes drove the field potentials in the thalamus and cortex.

### Statistical analyses

One-way repeated-measures ANOVA and paired-sample *t* test were performed in the 2 experiments respectively to evaluate the impact of stimulus intensity on nocifensive behavioral scores. Three-way repeated-measures ANOVA was conducted to assess the effect of the experimental factors (intensity [3 levels in Experiment 1: low, middle, and high; 2 levels in Experiment 2: low and high], hemisphere [2 levels: contralateral and ipsilateral to the stimulated side] and brain region [2 levels in Experiment 1: S1 and VPL; 4 levels in Experiment 2: S1, VPL, ACC, and MD]) on laser-evoked brain responses: (a) LFP responses in all brain regions (i.e., N1 amplitude and latency, LEP magnitude and GBO magnitude); (b) spike-firing rates in all brain regions. When significant, post hoc pairwise comparisons were performed using paired-sample *t* tests with Tukey’s correction. For the PCA results, in Experiment 1, a 1-way repeated-measures ANOVA was conducted to evaluate the impact of stimulus intensity on the amplitude of the thalamic EN component. Similarly, in Experiment 2, a 2-way repeated-measures ANOVA was used to assess the impact of stimulus intensity and brain region on the amplitude of the thalamic EN component. To examine the latency difference between the cortical N1 and thalamic EN, paired-sample *t* tests were performed in both experiments. For SFGC results, paired-sample *t* tests were conducted to compare the strength difference of information flow between the thalamus and cortex. Additionally, 1-way repeated-measures ANOVA was used to examine peak latency differences between the SFGC, thalamic EN, and cortical N1 in both experiments.

## Data Availability

The supporting data are available at ScienceDB (https://doi.org/10.57760/sciencedb.16406).
