# Supplementary material for: Deciphering Authentic Nociceptive Thalamic Responses in Rats
Source: Research (Wash D C). 2024 Apr 9;7:0348. doi: 10.34133/research.0348 (PMC11014087; doi:10.34133/research.0348)
Supplement: Supplementary 1 — Figs. S1 to S4 Tables S1 to S5 [file research.0348.f1.zip › Figure S1.pdf]

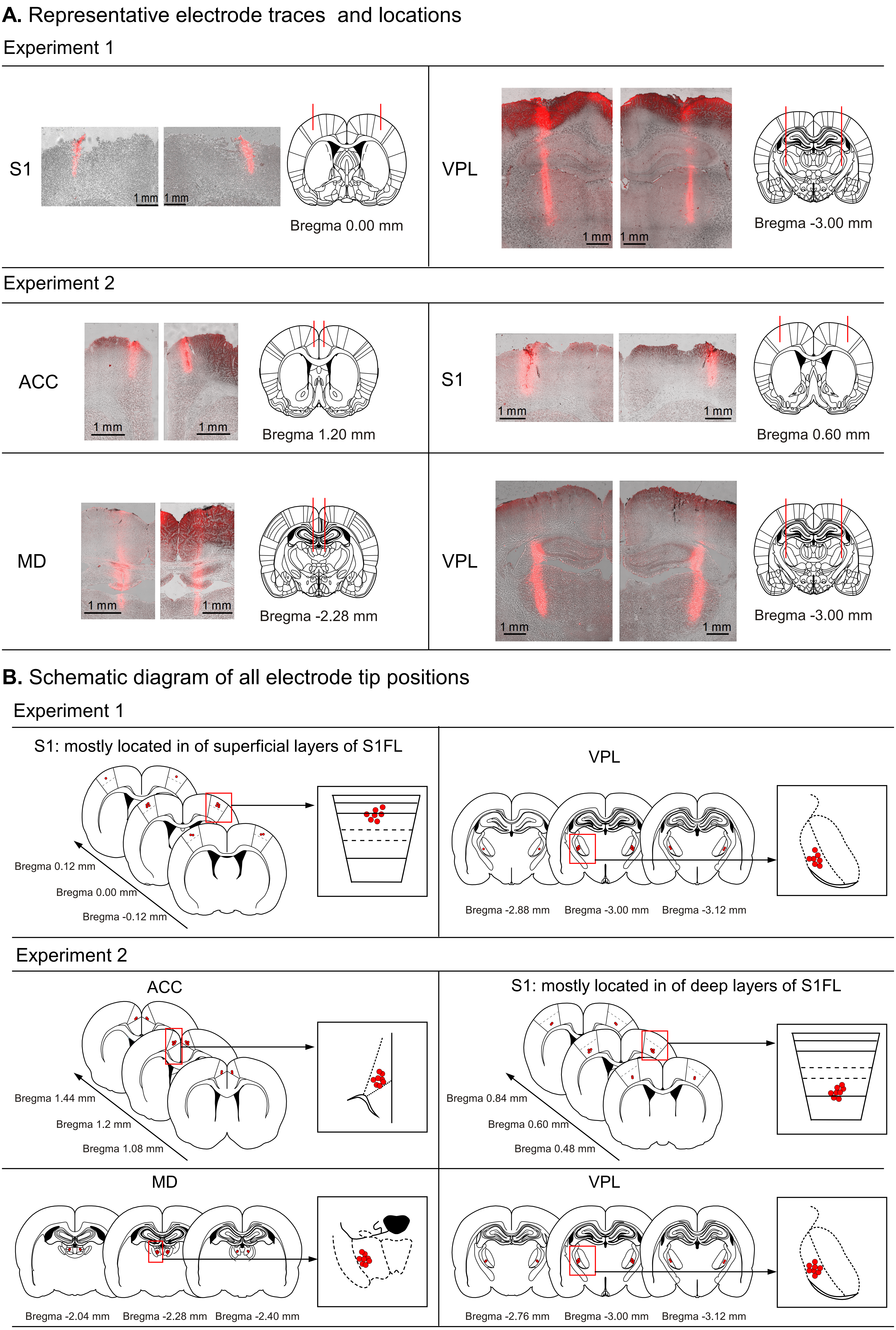
A. Representative electrode traces and locations
Experiment 1
S1
1 mm
1 mm
Bregma 0.00 mm
VPL
1 mm
1 mm
Bregma -3.00 mm
Experiment 2
ACC
1 mm
1 mm
Bregma 1.20 mm
S1
1 mm
1 mm
Bregma 0.60 mm
MD
1 mm
1 mm
Bregma -2.28 mm
VPL
1 mm
1 mm
Bregma -3.00 mm
B. Schematic diagram of all electrode tip positions
Experiment 1
S1: mostly located in of superficial layers of S1FL
Bregma 0.12 mm
Bregma 0.00 mm
Bregma -0.12 mm
VPL
Bregma -2.88 mm
Bregma -3.00 mm
Bregma -3.12 mm
Experiment 2
ACC
Bregma 1.44 mm
Bregma 1.2 mm
Bregma 1.08 mm
S1: mostly located in of deep layers of S1FL
Bregma 0.84 mm
Bregma 0.60 mm
Bregma 0.48 mm
MD
Bregma -2.04 mm
Bregma -2.28 mm
Bregma -2.40 mm
VPL
Bregma -2.76 mm
Bregma -3.00 mm
Bregma -3.12 mm
