# Supplementary material for: Deciphering Authentic Nociceptive Thalamic Responses in Rats
Source: Research (Wash D C). 2024 Apr 9;7:0348. doi: 10.34133/research.0348 (PMC11014087; doi:10.34133/research.0348)
Supplement: Supplementary 1 — Figs. S1 to S4 Tables S1 to S5 [file research.0348.f1.zip › Figure S2.pdf]

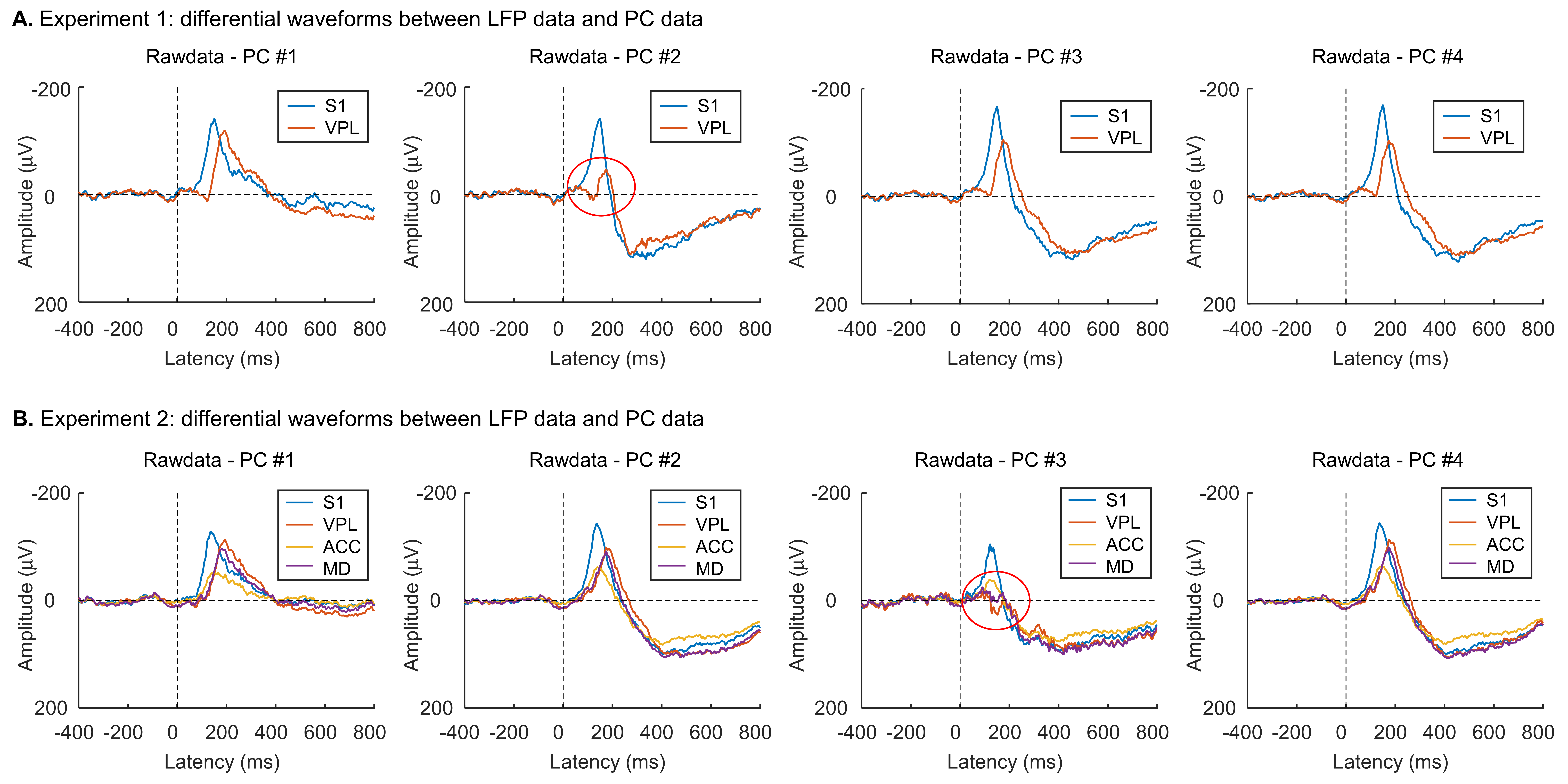
A. Experiment 1: differential waveforms between LFP data and PC data
Rawdata - PC #1
Rawdata - PC #2
Rawdata - PC #3
Rawdata - PC #4
S1
VPL
Amplitude (µV)
Latency (ms)
-200
0
200
-400 -200 0 200 400 600 800
B. Experiment 2: differential waveforms between LFP data and PC data
Rawdata - PC #1
Rawdata - PC #2
Rawdata - PC #3
Rawdata - PC #4
S1
VPL
ACC
MD
Amplitude (µV)
Latency (ms)
-200
0
200
-400 -200 0 200 400 600 800
