# Supplementary material for: Deciphering Authentic Nociceptive Thalamic Responses in Rats
Source: Research (Wash D C). 2024 Apr 9;7:0348. doi: 10.34133/research.0348 (PMC11014087; doi:10.34133/research.0348)
Supplement: Supplementary 1 — Figs. S1 to S4 Tables S1 to S5 [file research.0348.f1.zip › Figure S3.pdf]

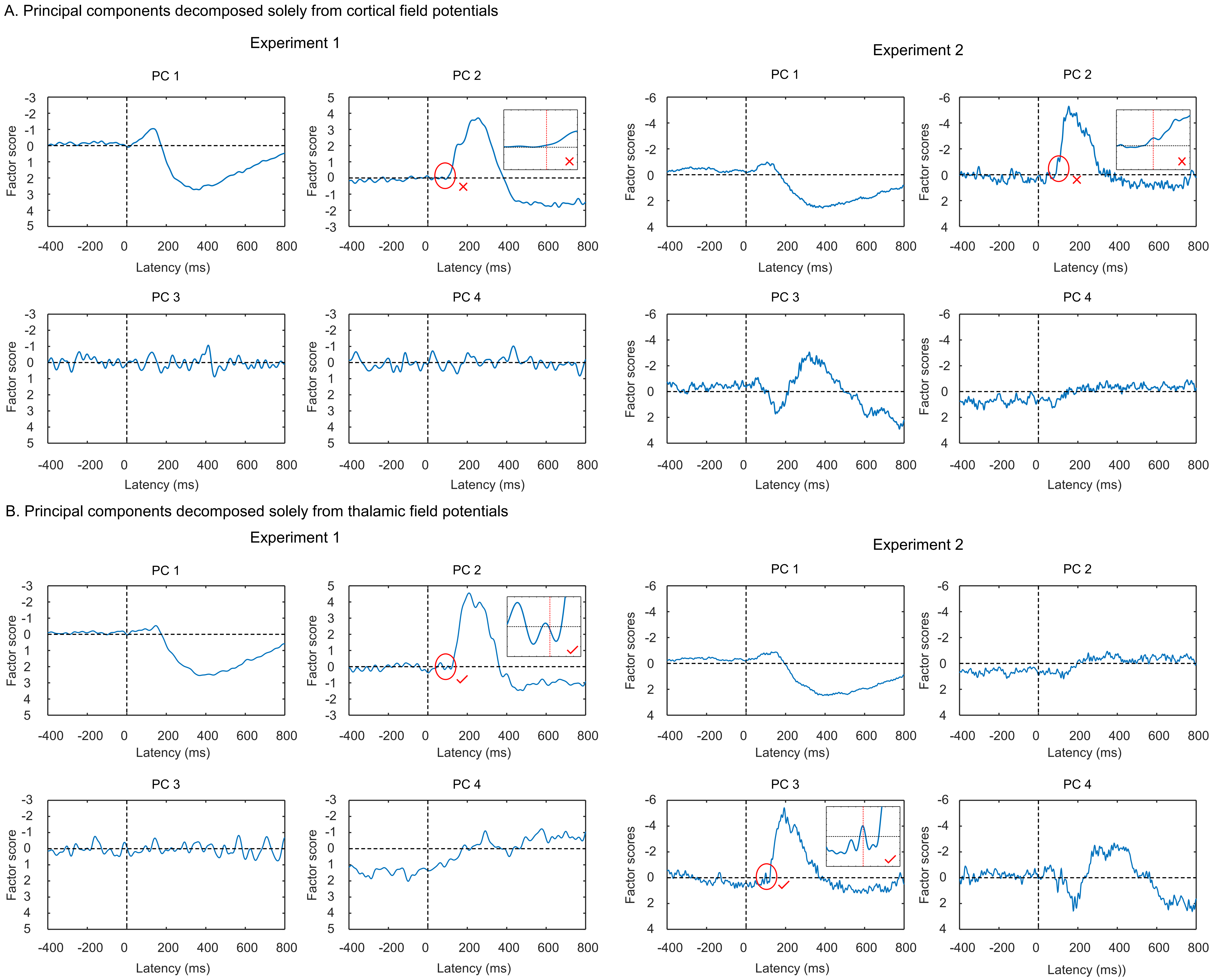
A. Principal components decomposed solely from cortical field potentials
Experiment 1
Experiment 2
PC 1
PC 2
PC 3
PC 4
Factor score
Factor scores
Latency (ms)
B. Principal components decomposed solely from thalamic field potentials
Experiment 1
Experiment 2
PC 1
PC 2
PC 3
PC 4
Factor score
Factor scores
Latency (ms)
Latency (ms))
