# Supplementary material for: Deciphering Authentic Nociceptive Thalamic Responses in Rats
Source: Research (Wash D C). 2024 Apr 9;7:0348. doi: 10.34133/research.0348 (PMC11014087; doi:10.34133/research.0348)
Supplement: Supplementary 1 — Figs. S1 to S4 Tables S1 to S5 [file research.0348.f1.zip › Figure S4.pdf]

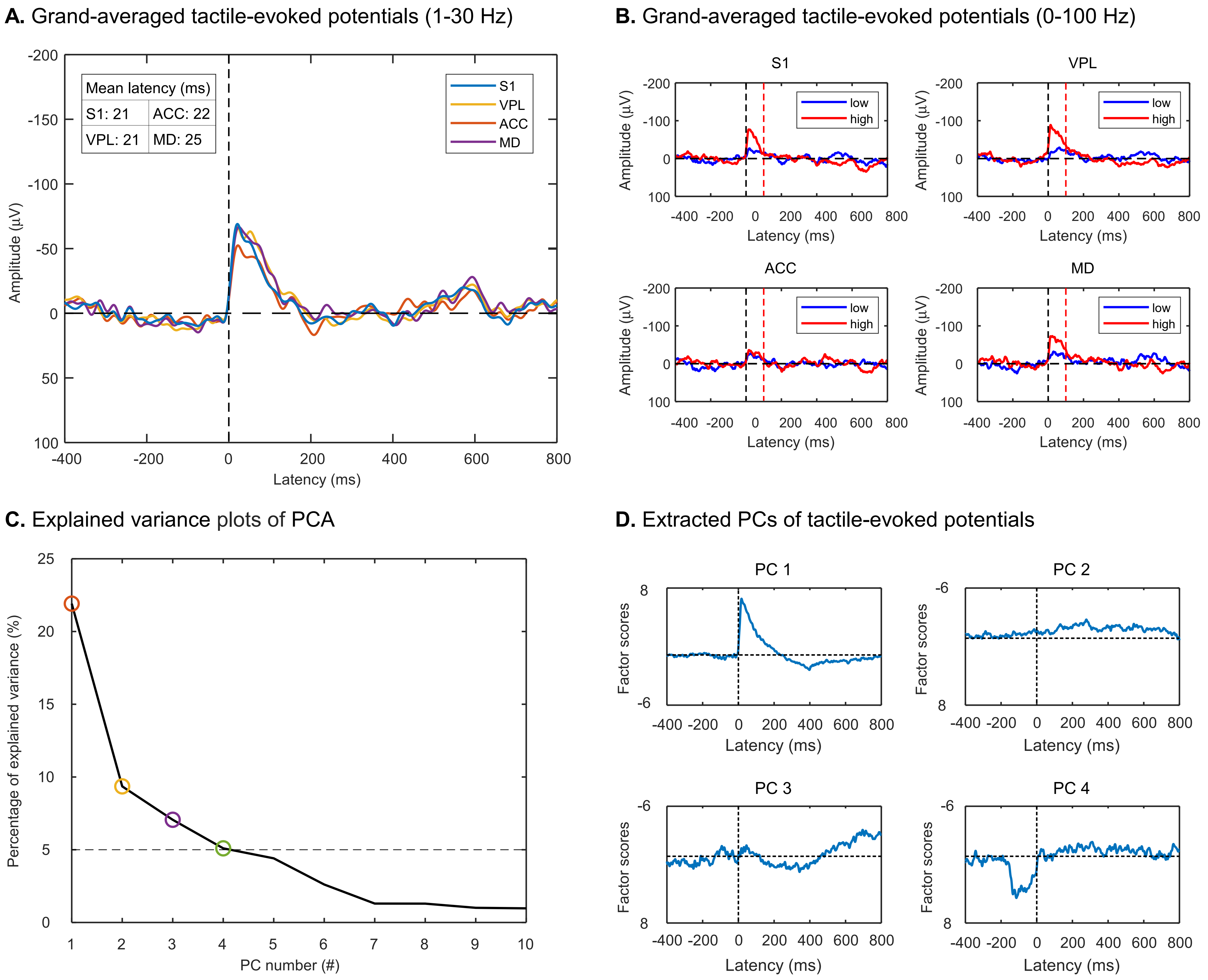
A. Grand-averaged tactile-evoked potentials (1-30 Hz)
Mean latency (ms)
S1: 21
ACC: 22
VPL: 21
MD: 25
S1
VPL
ACC
MD
Amplitude (μV)
-200
-150
-100
-50
0
50
100
-400
-200
0
200
400
600
800
Latency (ms)
B. Grand-averaged tactile-evoked potentials (0-100 Hz)
S1
VPL
ACC
MD
low
high
Amplitude (μV)
Latency (ms)
C. Explained variance plots of PCA
Percentage of explained variance (%)
25
20
15
10
5
0
1
2
3
4
5
6
7
8
9
10
PC number (#)
D. Extracted PCs of tactile-evoked potentials
PC 1
PC 2
PC 3
PC 4
Factor scores
8
-6
-400 -200 0 200 400 600 800
Latency (ms)
